# Hypofractionation and Concomitant Boost in Ductal Carcinoma In Situ (DCIS): Analysis of a Prospective Case Series with Long-Term Follow-Up

**DOI:** 10.3390/life12060889

**Published:** 2022-06-14

**Authors:** Domenico Cante, Marina Paolini, Cristina Piva, Edoardo Petrucci, Lorenzo Radici, Silvia Ferrario, Guido Mondini, Silvia Bagnera, Maria Rosa La Porta, Pierfrancesco Franco

**Affiliations:** 1 Department of Radiation Oncology, ASL TO4, Ivrea Community Hospital, 10015 Ivrea, Italy; dcante@aslto4.piemonte.it (D.C.); mpaolini@aslto4.piemonte.it (M.P.); cpiva@aslto4.piemonte.it (C.P.); sferrario@aslto4.piemonte.it (S.F.); mlaporta@aslto4.piemonte.it (M.R.L.P.); 2 Department of Medical Physics, ASL TO4, Ivrea Community Hospital, 10015 Ivrea, Italy; epetrucci@aslto4.piemonte.it (E.P.); lradici@aslto4.piemonte.it (L.R.); 3 Department of Surgery, ASL TO4, Ivrea Community Hospital, 10015 Ivrea, Italy; gmondini@aslto4.piemonte.it; 4 Department of Diagnostic Imaging, ASL TO4, Ivrea Community Hospital, 10015 Ivrea, Italy; sbagnera@aslto4.piemonte.it; 5 Department of Translational Medicine (DIMET), ‘Maggiore della Carità’ University Hospital, University of Eastern Piedmont, 28100 Novara, Italy

**Keywords:** ductal carcinoma in situ, DCIS, hypofractionated radiotherapy, hypofractionation

## Abstract

We previously reported on a cohort of breast cancer patients affected with ductal carcinoma in situ (DCIS) that were treated with breast conservative surgery and hypofractionated whole-breast radiotherapy with a concomitant boost to the lumpectomy cavity. We now report on the long-term results of the oncological and toxicity outcomes, at a median follow-up of 11.2 years. We also include an analysis of the predictive factors for local recurrence (LR). Eighty-two patients with long-term observation were considered for this report. All received hypofractionated post-operative radiotherapy with a concomitant boost (45 Gy/20 fractions to the whole breast and 50 Gy/20 fractions to the lumpectomy cavity). We report on LC rates at 5 and 10 years, overall survival (OS), and breast-cancer-specific survival (BCSS), employing the Kaplan–Meier method. Cox proportional regression analysis was used to determine the role of selected clinical parameters on the risk of local recurrence, by the univariate and multivariate models. After a median follow-up of 11.2 years (range 5–15 years), 9 pts (11%) developed LR. The LR rates at 5 years and 10 years were 2.4% and 8.2%, respectively. The 5- and 10-year overall survival rates were 98.8% and 91.6%, respectively. The 5- and 10-year breast-cancer-specific survival rates were 100.0% and 99.0%. Late skin and subcutaneous toxicities were generally mild, and cosmetic results were good–excellent for most patients. For the univariate regression analysis, ER positive status (HR; 95% CI, *p* = 0.021), PgR positive status (HR; 95% CI, *p* = 0.012), and the aggregate data of positive hormonal status (HR; 95% CI, *p* = 0.021) were inversely correlated to LR risk. Conversely, a high tumor grade (G3) was directly correlated with the risk of LR (HR; 95% CI, *p* = 0.048). For the multivariate regression analysis, a high tumor grade (G3) confirmed its negative impact on LR (HR 0.40; 95% CI 0.19–0.75, *p* = 0.047). Our long-term data demonstrate hypofractionated whole-breast radiotherapy with a concomitant boost to be feasable, effective, and tolerable. Our experience suggests positive hormonal status to be protective with respect to LR risk. A high tumor grade is a risk factor for LR.

## 1. Introduction

In recent years, ductal carcinoma in situ (DCIS) of the breast is most frequently diagnosed in asymptomatic patients, after screening mammography for microcalcifications, accounting for the extremely favorable survival outcomes [1]. Even if no randomized trial compared overall (OS) and disease-free (DFS) survival in DCIS patients undergoing breast conservation or a mastectomy, breast-conserving surgery (BCS) and post-operative treatments are considered as oncologically safe approaches in this setting, specifically for patients harboring limited lesions [2]. Hence, post-operative whole-breast irradiation (WBI) after BCS is presently deemed as a standard option for most DCIS cases [1]. This is supported by the data derived from the clinical trials investigating the role of radiotherapy after BCS (NSABP B17, EORTC 10853, SweDCIS, UK/ANZ), and by a meta-analysis demonstrating a 15% absolute 10-year risk reduction at 10 years, for both subsequent ipsilateral DCIS (DCIS) and ipsilateral invasive-breast-cancer lesions, with the addition of WBI [3].

Historically, as with invasive carcinomas, the standard radiation approach used in all WBI trials in DCIS was 50 Gy administered in 25 fractions over 5 weeks (CF-WBI).

Three recent prospective trials explored hypofractionated regimens in patients with DCIS. In the RTOG 9804 trial, about 10% of patients were treated according to the Canadian hypofractionation schedule used for invasive breast cancer [4]. In the Danish Breast Cancer Group hypofractionation trial, a total of 246 patients were treated with the UK START B 15-fraction hypofractionated regimen [5]. In the BIG 03-07/TROG 07-01 trial, patients enrolled in two of the experimental arms (563 individuals) received hypofractionated WBI (HF-WBI) (42.5 Gy/16 fractions over 3 weeks) as part of their treatment strategy [6]. The aforementioned studies and other retrospective reports suggest a substantial equivalence in terms of local control rates, with similar toxicity and cosmesis outcome, for HF-WBI compared to CF-WBI [7,8,9,10,11,12,13,14]. Recent Italian and Korean propensity score-matched analyses from national multicenter cohorts corroborate these findings [15,16]. The role of a boost dose to the lumpectomy cavity for DCIS after BCS is debated. A meta-analysis of observational studies showed a boost to reduce the risk of LRs in the case of positive margins, but a subsequent multicenter retrospective trial failed to confirm this finding [11,17,18]. In the BIG 03-07/TROG 07-01, a risk reduction of more than half was observed at 5 years, for patients receiving a boost dose to the tumor bed. The benefit was consistent across subgroups [19].

Other patient-, tumor-, and treatment-related characteristics were observed to have an impact on clinical outcome, such as age, tumor grade, comedo-type necrosis, close or involved margins, estrogen (ER) and progesteron (PgR) receptor status, and administration of endocrine therapy [14,15,16,17].

We previously reported on a cohort of DCIS patients treated with BCS and HF-WBI with a concomitant boost to the tumor bed [10]. After a median follow-up of 48 months, no local recurrence was observed. This approach proved to be safe and effective, providing excellent local control rates, consistent cosmetic results, and mild toxicity [10].

We herein present long-term results on clinical outcomes for DCIS patients, together with an analysis of predictive factors for local recurrence (LR) in this setting.

## 2. Materials and Methods

Between 2005 and 2012, a total of 82 patients affected with DCIS and submitted to BCS received HF-WBI with a concomitant boost to the lumpectomy cavity at our institution.

### 2.1. Cohort Characteristic

Patients had a histological diagnosis of breast DCIS, previous BCS (lumpectomy/quadrantectomy), negative resection margins (>3 mm), and a pathological pTis stage, according to the American Cancer staging system (AJCC-UICC; 6a edition); positive surgical margins, previous thoracic radiation, second synchronous primary tumor, and age >80 years were considered non-eligibility criteria.

### 2.2. Radiotherapy Treatment Schedule

All patients received 45 Gy/20 fractions (2.25 Gy per day) and 50 Gy/20 fractions (2.5 Gy per day), respectively, planned on the whole breast and tumor bed (identified by surgical clips). Both target volumes were integrated into the same treatment plan and, therefore, the concomitant delivery in the same treatment session of a different dose per fraction was given with 6 MV photon fields. We previously reported details about contouring, planning, and treatment delivery for both invasive carcinoma and DCIS [10,20,21,22,23].

### 2.3. ER ang PgR Status

For ER and PgR status, two categories (negative and positive) were considered according to a widely used 10% cut-off value (for both ER and PgR) [24]. Positive hormonal status (HS) was defined as positive ER and/or PgR status.

### 2.4. Other Pathological Characteristics

Breast cancer was classified according to the histological type and staged following the TNM classification of malignant tumors [25]. Histological tumor grading was assessed according to Elston and Ellis [26]. For the variable tumor grade, 3 categories were considered during the analysis: G1, G2, and G3. Further analysis was then performed grouping patients into 2 grading categories: G1–G2 vs. G3. All other histopathologic features (subtype comedo, necrosis, microcalcifications, and multifocal disease) were evaluated as categorical variables (either present or absent).

### 2.5. Follow-Up and Toxicity

In the follow-up period, patients were evaluated at 3 months and 6 months, after treatment, and twice a year thereafter for 5 years, then annually. Surveillance included a clinical examination at every time point, plain chest X-ray, mammography, and complete blood cell count once a year. Other radiological examinations were performed as needed. Local recurrence was recorded. The update on late toxicity and cosmetic outcome was based on the Common Terminology Criteria for Adverse Events, version 3.0, for late effects, and the Harvard criteria, respectively.

### 2.6. Statistical Analysis

Standard descriptive statistics was used to describe the main individual characteristics for both patients and tumors. LR was defined as histologically proven DCIS or an invasive disease in the ipsilateral breast. Kaplan–Meier analysis was used to estimate 5- and 10-year local relapse, OS, and BCS. Differences between groups were evaluated by the log-rank test. The follow-up time was calculated from the date of the first surgery to the date of the last follow-up visit, or to the date of LR or death. Cox proportional regression analysis was used to determine the role of selected parameters on the risk of LR by univariate models and then by multivariate models, including those parameters found to be statistically significant at univariate analysis. The risk of LR was calculated as a hazard ratio (HR) with corresponding 95% confidence intervals (95% CI). *p*-values less than 0.05 were considered statistically significant. All statistical tests were performed using the IBM SPSS Statistics software (Statistical Package for Social Science, version 22).

## 3. Results

The 82 patients analyzed in the present report have a minimum observation time of 60 months. Patient, tumor, and treatment characteristics are summarized in Table 1. The median age at diagnosis was 60.5 years (range 37–78 years). Most of the patients were in a postmenopausal status (84.1%). They all underwent complete surgical excision with negative margins. A sentinel lymphnode biopsy was performed in 30 patients (36.6%). ER and/or PgR were positive in 68 patients (82.9%). Endocrine therapy (either tamoxifen or aromatase inhibitor) was given to 89.7%. The choice not to offer endocrine therapy was related to age >70 years or the patient’s preference. Only for 55 patients, a Ki-67 assessment was available, and 42 patients had a low proliferation index. Tumors were mainly low-grade (G1–G2 63.4%); necrosis and the comedo subtype was reported in 39% and 51.2% of patients, respectively. Few tumors were multifocal (26.8%), while microcalcifications were presents in 34 patients.

### 3.1. Clinical Outcomes

**Figure 1:**
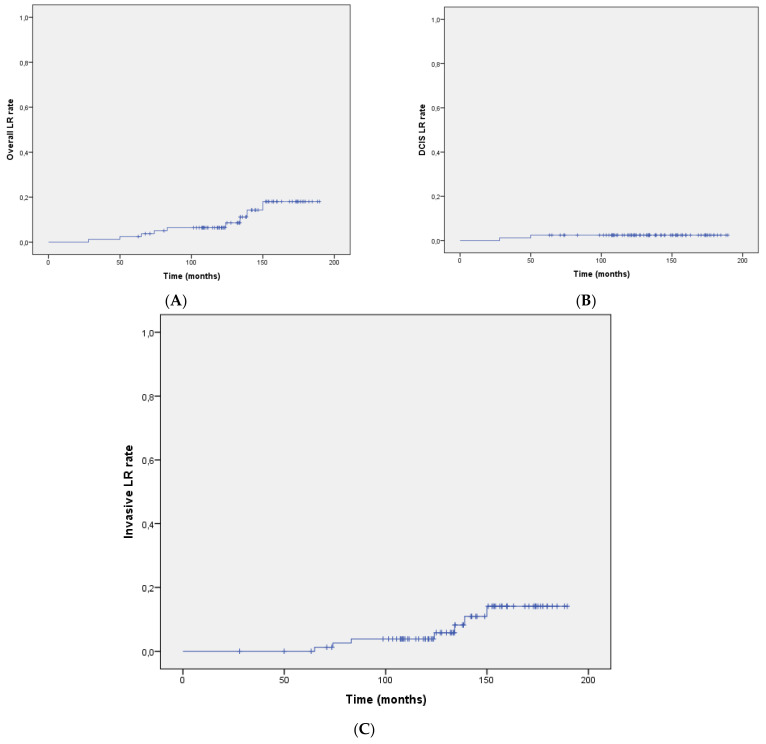
(**A**) Cumulative local recurrence rate (both invasive and in situ). (**B**) Cumulative in situ local recurrence rate. (**C**) Cumulative invasive local recurrence rate.

After a median follow-up of 11.2 years (range 5–15 years), nine patients (11%) developed LR, of which seven were an invasive disease (77.8%) and two were DCIS (22.2%). For four out of nine (44.4%) patients, the relapse occurred in the same quadrant in which the original DCIS was diagnosed. Median time to recurrence was 7.1 years (range 2.42–12.70 years). The LR rates at 5 years and 10 years were 2.4% (95% CI 0.7–4.1) and 8.2% (95% CI 4.9–11.5), respectively. LR-rate Kaplan–Meier curves (for both DCIS and invasive recurrences) are shown in Figure 1A–C. Amongst the nine patients who experienced an LR, seven (77.8%) were salvaged with a mastectomy and two had a second conservative surgery. After surgery, two patients received chemotherapy, four received endocrine therapy, and three patients were observed clinically.

Ten patients (12.2%) died during the follow-up period. The 5- and 10-year OS rates were 98.8% and 91.6%, respectively. Only one death was related to breast cancer, with a patient developing distant metastases. Five deaths were from other malignant causes, three from primary gatrointestinal cancer, and one from either cerebral or hematological malignancies. There was one death caused by a cerebral vascular accident, one from post-operative complications of an orthopedic surgery, and two from senescence. The 5- and 10-year breast-cancer-specific survival rates were 100% and 99%, respectively.

We observed seven contralateral breast cancers (8.5%), and two out of seven (28.6%) were DCIS and five out of seven (71.4%) were invasive breast cancer; five out of seven (71.4%) of them occurred less than 5 years after the diagnosis of primary DCIS. All but one patient with contralateral disease were given adjuvant endocrine therapy after the first diagnosis of DCIS.

### 3.2. Predictive Factors

The results of univariate and multivariate analyses for LR are summarized in Table 2.

**Figure 2:**
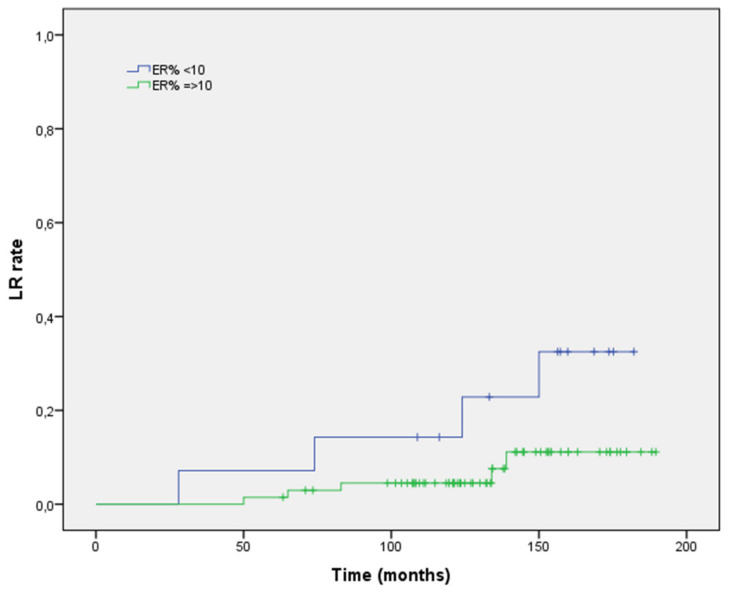
Local recurrence rate stratified according to estrogen-receptor expression.

**Figure 3:**
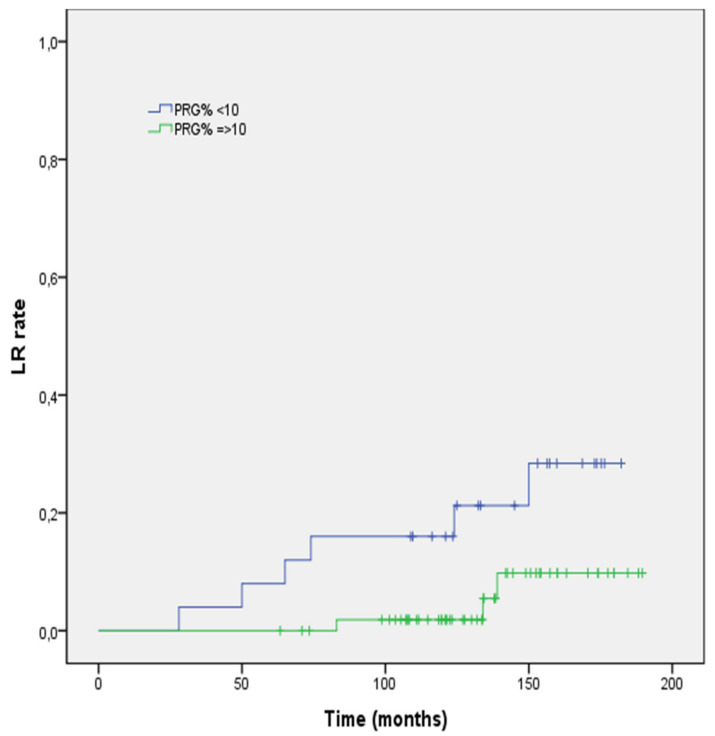
Local recurrence rate stratified according to progester-receptor expression.

**Figure 4:**
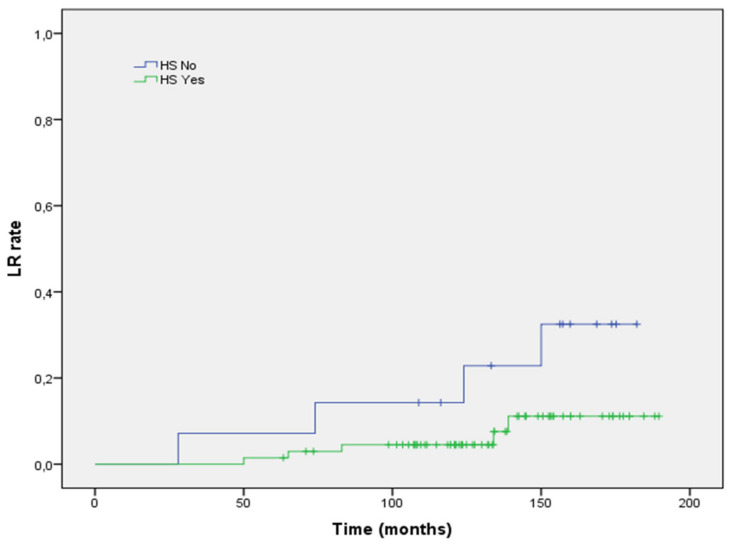
Local recurrence rate stratified according to tumor grading.

At univariate regression analysis, ER positive status (HR 0.32; 95% CI 0.21–0.72, *p* = 0.021; Figure 2), PgR positive status (HR 0.55; 95% CI 0.32–0.96, *p* = 0.012; Figure 3), and aggregate data of positive HS (HR 0.32; 95% CI 0.21–0.72, *p* = 0.021; Figure 4) were inversely correlated to LR risk. Conversely, tumor grade G3 (vs. G1–G2) was directly correlated with LR risk (HR 0.47; 95% CI 0.28–0.96, *p* = 0.048). None of the remaining histopathologic factors examined showed a significant impact on the LR rate at univariate analysis. With respect to patient-related factors, neither age (*p* = 0.093) nor post-menopausal status (*p* = 0.128) had a significant impact on LR. Receiving post-operative endocrine had no correlation with LR risk (*p* = 0.46). At multivariate regression analysis, tumor grade G3 (vs. G1–G2) confirmed its negative impact on LR (HR 0.40; 95% CI 0.19–0.75, *p* = 0.047).

### 3.3. Toxicity and Cosmetic Outcome

Late skin and subcutaneous toxicities were generally mild, with only 1% of patients experiencing G3 events. No major lung and heart toxicity was detected. Cosmetic results were excellent in 50% of patients, good in 40%, fair in 8%, and poor in 2%.

Grade 1–2 late effects were mostly related to skin and subcutaneous tissues, namely fibrosis and induration, skin atrophy, teleangiectasia, and hyperpigmentation. The cumulative rate of grade 1–2 events was 9.2%.

During the follow-up, a case of radiation-induced breast angiosarcoma was observed, which was diagnosed 10 years after the previous radiotherapy course. The patient underwent neoadjuvant chemotherapy as well as a subsequent mastectomy and subsequent chemotherapy.

## 4. Discussion

In our cohort of patients affected with breast DCIS and submitted to HF-WBI with a concomitant boost to the lumpectomy cavity, an LR rate of 11% was observed after 10 years of follow-up. This is in line with other reports available in the literature. At a mean follow-up of about 7 years, an 11% risk of ipsilateral recurrence was reported in the analysis of the SEER database [27]. In the population-based Munich Cancer Registry, Corradini et al. described a cumulative incidence of ipsilateral in-breast tumor recurrence of 13.6% at 10 years [28]. A slightly lower rate of local relapse was reported in an NSABP B-17 trial, with a 15-year cumulative incidence of ipsilateral recurrences of 8%, and the same results was documented by an EORTC 10853 trial, with a 8% incidence of LR after a 10-year follow-up [27,29]. More recently, Meattini et al. collected clinical data from nine Italian centers on 1072 women with diagnosis of DCIS and reported a 6.3% rate of LR, at a median follow-up of 8.4 years [13].

Different retrospective series, employing HF-WBI, reported similar LR rates compared with CF-WBI. A Canadian group reported on more than 1500 patients, after a median follow-up of almost 10 years; hypofractionation was not associated with an increased risk of LR on multivariate analysis. Local recurrence rates were 13% for CF-WBI and 10% for HF-WBI, respectively [7]. Isfahanian et al. reviewed radiotherapy management for DCIS and its impact on LR: the 5-year LR-free survival rate was 94% for the HF-WBI group vs. 91% for the CF-WBI group (*p* = 0.80) [8]. The most recent comparison publication is by De Rose et al. [10]. This analysis included more than 500 patients from four Italian centers treated with CF-WBI 50 Gy/25 fractions or HF-WBI 40.5 Gy/15 fractions. With 10 years of median follow-up, no correlation was observed between the type of radiotherapy schedule and LR-free survival. A 2015 meta-analysis of observational studies also confirmed that hypofractionation is a safe and effective option for DCIS. However, the authors concluded that the level of evidence for these observations is in the very low range, so randomized trials are necessary to confirm the results [11].

Prospective data are available in this setting. The long-term results of a Danish Breast Cancer Group (DBCG) HYPO trial, including 246 patients with DCIS, have been recently published. This study compared hypofractionated and conventional schemes, in term of breast induration (primary end point), and no significant difference was reported. The 9-year locoregional recurrence risk was low in both groups (3% and 3.3%, respectively), with a median follow-up time of 7.26 years [29].

An international multicenter randomized phase 3 trial (BIG 03–07/TROG 07.01), comparing CF-WBI ± boost (10 Gy in fractions of 2 Gy) to HF-WBI ± boost (10 Gy in fractions of 2.5 Gy) in non-low-risk DCIS, is ongoing. Data about cosmetic outcomes and health-related quality of life at 2 years were reported [30,31]. More recently, results about LR were reported, with no difference between the two regimens at a median follow-up of 6 years. 

The results of studies addressing the eventual clinical benefit of administering a boost to the tumor bed are conflicting.

A meta-analysis of observational studies showed a boost to reduce the risk of LR in case of positive margins [11], with low level of evidence. Cambra et al. reported results of a retrospective, multicentre study of 622 patients diagnosed with pure DCIS, to evaluate the role of a boost on local control. Although boost radiotherapy was more common in patients with high-risk disease, it did not reduce the risk of ipsilateral breast tumor recurrence [17]. The only significant independent prognostic factors at multivariate analysis were tumor size and re-excision.

Moran et al. reported the results of a retrospective analysis from 10 academic institutions in the United States, Canada, and France [11]. A total of 4131 patients was included, with a median follow-up of 9 years and a median boost dose of 14 Gy. The authors conclude that a radiotherapy boost dose to the lumpectomy cavity may decrease LR across all DCIS.

In 2016 Cutuli et al. reported on local recurrence rates in patients with ductal carcinoma in situ, treated by BCS and WBI with or without a boost and/or tamoxifen, and compared different therapeutic options in two European countries. Data were collected on 819 patients, with a median follow up of 90 months, observing 51 LRs (6.2%). The use of boost did not influence local recurrence rates, whereas hormonal treatment was the most significant protective factor [16].

In this setting, as well as for of the ongoing Bonbis trial, the results are pending for comparing a boost of 16 Gy vs. no boost [32].

Different risk factors were identified for local recurrence; some are related to the patient, some to the tumor, and others to the treatment [29,30,31,32,33]. Wang et al., identified comedonecrosis, focality, margin status, method of detection, tumor grade, and tumor size as risk factors for LR [34]. Zhang et al. confirmed significant associations with LR for positive margin status andthe diagnosis method, but did not for nuclear grade, comedonecrosis, tumor size, multifocality, estrogen receptor- and progesterone receptor-status, or HER2/neu positivity [35].

In our cohort, the significant risk factor for LR on the univariate analysis was grade (G3 vs. G1–G2), while positive ER status, positive PgR status, and the aggregate data of positive hormonal status were found to be protective for LR. At multivariate regression analysis, tumor grade G3 vs. G1–G2 confirmed its negative impact on LR.

With regard to nuclear grade, Cutuli et al. reported high nuclear grade and lack of tamoxifen as the most powerful predictors of LR. Particularly, grading represented the most predictive histopathological factor for LR, with a 10.5% rate vs. a 5% rate for high and low/intermediate grade, respectively (*p* = 0.0051) [16]. In the EBCTCG meta-analysis, radiotherapy decreased L210457R rates by 18% (from 30% to 12%) and 10% (from 26% to 16%) in low- and intermediate-/high-grade lesions [1]. In a more recent publication by De Rose et al., high grade (HR 1.85, 95% CI 1.23–2.77; *p* = 0.003) and positive margins (HR 1.99, 95% CI 1.06–3.73; *p* = 0.031) were correlated with worse LRFS [15].

Several studies have shown the impact of hormonal status on local outcome. Meattini et al. retrospectively analyzed a series of almost 300 patients with DCIS to evaluate the patterns of recurrence. At multivariate analysis, positive ER status shows a protective role (*p* = 0.003) [36]. This finding was then confirmed on a series of 1072 women affected by DCIS, with ER positivity found to be a significantly favorable feature (*p* = 0.045) [32].

In our experience, endocrine therapy was not shown to be a protective factor for LR. In the literature, there are several studies showing an advantage for patients with positive HS [16,22,23]. The already mentioned study by Cutuli et al. reported a lack of tamoxifen as a predictor of LR, with a 2.85 (95% CI: 1.42–5.72, *p* = 0.04) odds ratio estimate [16]. The value of tamoxifen therapy in addition to RT was also tested in the UK/ANZ trial; at 12 years of follow-up, tamoxifen reduced ipsilateral DCIS recurrent (0.70, 0.51–0.86; *p* = 0.03) and contralateral tumors (0.44, 0.25–0.77; *p* = 0.005) [37].

The rationale for adjuvant hormonal therapy in DCIS is dual: to reduce the risk of LR, and to reduce the risk of new primary cancers in the same or contralateral breast [38]. Probably given the small number of events in our cohort, this positive effect did not emerge. Indeed, we recorded seven contralateral breast cancers and five of them occurred less than 5 years after the diagnosis of primary DCIS: all but one of these five patients took hormone therapy for the first diagnosis of DCIS. There are no available data regarding compliance with hormone treatment.

In our study, none of the main patient-related characteristics was shown to be correlated with the risk of local recurrence, especially age and postmenopausal status. This is in disagreement with numerous of the literature studies. Meattini et al. reported a favorable prognostic role of the postmenopausal state on LR occurrence [29]. Poulakaki et al. reported older age at diagnosis and postmenopausal status to be associated with better prognosis [34].

Given the homogeneity of treatment in our study, no conclusion can be drawn regarding the influence of treatment-related factors.

Finally, the analysis of late toxicity and post-RT cosmesis shows that HF-WBI with a concomitant boost to the lumpectomy cavity offers excellent results, even with longer follow-up, due to robust results in terms of dose conformity and homogeneity, achievable with both 3D-conformal and intensity-modulated techniques, as previously demonstrated [39,40,41].

This is in line with the other literature data regarding HF-WBI, especially with the recent results of the HYPO trial and the preliminary results of the TROG 07.01 trial [5,6]. Whereas, concerning a boost, preliminary results of the TROG 07.01 trial report a deterioration of cosmetic outcomes in case of a tumor bed boost, but in the study protocol the dose administered was 16 Gy, sequentially, to WBI [6]. In our study, although a boost was administered concomitantly with a mild hypofractionation (2.5 Gy/die), the total dose was 5 Gy, with the total dose of 50 Gy, finally, to the tumor bed.

The present study has some limitations, including the slenderness of the sample size, its retrospective nature, and a lack of a control group that was treated with conventional fractionation and a sequential boost. Nevertheless, it reports on long-term results for both local control as well as the survival and toxicity outcomes of a cohort of DCIS breast-cancer patients, treated with WBI and a concomitant boost to the lumpectomy cavity. Few studies with a long observation time are present in the literature, and this study adds up the current knowledge, in this sense.

## 5. Conclusions

Hypofractionation is a frequently employed strategy to perform WBI after BCS in DCIS. Our long-term data demonstrate safety, efficacy, and tolerability. Our experience suggests that ER positive status, PgR positive status, and aggregate data of positive hormonal status were inversely correlated to LR risk. Conversely, a high tumor grade was a major risk factor for LR. In the era of personalized medicine, prospective data are necessary to estimate the benefit of adjuvant therapy for DCIS and to further characterize reliable risk-group stratification [42].

## Figures and Tables

**Table 1:** Patient, tumor, and treatment characteristics.

Patient, Tumor, and Treatment Characteristcs		
Age Groups (Years)	N° Patients	%
<40	2	2.4
40–60	39	47.6
>60	41	50
Menopausal state		
Premenopausal	13	15.9
Postmenopausal	69	84.1
Patological nodal stage		
pN0	35	42.7
pNX	47	57.3
Grading		
G1	15	18.3
G2	37	45.1
G3	30	36.6
Subtype comedo		
Yes	42	51.2
No	40	48.8
Necrosis		
Yes	32	39
No	50	61
Microcalcifications		
Yes	34	41.5
No	48	58.5
Multifocality		
Yes	22	26.8
No	60	73.2
Ki-67%		
<20	42	76.4
>40	13	23.6
NA	27	-
ER%		
<10	14	17.1
≥10	68	82.9
Prg%		
<10	25	30.5
≥10	57	69.5
Hormonal status		
Yes	68	82.9
No	14	17.1
Hormone Therapy *		
Yes	61	89.7
No	7	10.3

* Total of 68 patients with positive hormonal-status disease.

**Table 2:** Prognostic factors.

Prognostic Factor			
Univariate Analysis	*p*-Value	β	HR (95% C.I.)
Age groups	0.093		
Menopausal state	0.128		
Grading	0.097		
**Grading: G1–G2 vs. G3**	**0.048**	−0.75	0.47 (0.28–0.96)
Subtype comedo	0.091		
Necrosis	0.650		
Microcalcifications	0.129		
Multifocality	0.802		
Ki 67%	0.913		
**ER%**	**0.021**	−1.55	0.32 (0.21–0.72)
**Prg%**	**0.012**	−0.65	0.55 (0.32–0.96)
**Hormonal status**	**0.021**	−1.55	0.32 (0.21–0.72)
Hormone Therapy *	0.460		
Multivariate analysis			
**Grading: G1–G2 vs. G3**	**0.047**	−0.99	0.40 (0.19–0.75)
ER%	0.970		
Prg%	0.222		
Hormonal status	0.970		

* Assessed on the 68 patients receiving hormonal therapy.

## Data Availability

Data are available upon request by contacting the corresponding author.
